# *OsHsfB4b* Confers Enhanced Drought Tolerance in Transgenic Arabidopsis and Rice

**DOI:** 10.3390/ijms231810830

**Published:** 2022-09-16

**Authors:** Yan Zhang, Chen Wang, Changyu Wang, Liu Yun, Linhu Song, Muhammad Idrees, Huiying Liu, Qianlong Zhang, Jingyu Yang, Xu Zheng, Zhiyong Zhang, Jie Gao

**Affiliations:** 1 College of Life Sciences, Neijiang Normal University, Neijiang 641004, China; 2 State Key Laboratory of Wheat and Maize Crop Science, and Center for Crop Genome Engineering, Longzi Lake Campus, College of Agronomy, Henan Agricultural University, Zhengzhou 450046, China

**Keywords:** rice, *Hsf*, genome-wide analysis, expression profiles, drought stress

## Abstract

Heat shock factors (Hsfs) play pivotal roles in plant stress responses and confer stress tolerance. However, the functions of several *Hsfs* in rice (*Oryza sativa* L.) are not yet known. In this study, genome-wide analysis of the *Hsf* gene family in rice was performed. A total of 25 *OsHsf* genes were identified, which could be clearly clustered into three major groups, A, B, and C, based on the characteristics of the sequences. Bioinformatics analysis showed that tandem duplication and fragment replication were two important driving forces in the process of evolution and expansion of the *OsHsf* family genes. Both *OsHsfB4b* and *OsHsfB4d* showed strong responses to the stress treatment. The results of subcellular localization showed that the OsHsfB4b protein was in the nucleus whereas the OsHsfB4d protein was located in both the nucleus and cytoplasm. Over-expression of the *OsHsfB4b* gene in Arabidopsis and rice can increase the resistance to drought stress. This study provides a basis for understanding the function and evolutionary history of the *OsHsf* gene family, enriching our knowledge of understanding the biological functions of *OsHsfB4b* and *OsHsfB4d* genes involved in the stress response in rice, and also reveals the potential value of *OsHsfB4b* in rice environmental adaptation improvement.

## 1. Introduction

As sessile organisms, plants cannot escape from a disadvantageous environment so they have evolved their developmental plasticity to optimize their growth and development [1]. Under harsh conditions, complex stress regulation and response networks have been developed in plants [2]. The response of plants to various stresses is mainly through the sensing and transduction of stress signals through signal elements, resulting in the expression of a large number of stress-related genes and the synthesis of various functional proteins [3]. Many physiological and biochemical changes occur in response and through adaptation to adverse environmental conditions. Many functional proteins, such as osmoregulatory proteins, ion channel proteins, transporters, antioxidants, and detoxifying proteins, are highly expressed under stress, and these functional proteins are largely regulated by specific transcription factors [4,5]. Among these plant transcription factors, Hsfs heat shock factors (Hsfs) are the terminal components of the signal transduction chain that are involved in various abiotic stress responses, and are essential for the maintenance of protein homeostasis as well as during normal growth conditions [6,7,8]. The basic structure and mode of promoter recognition of Hsfs are conserved throughout the prokaryotes and the eukaryotes kingdom [9,10,11]. 

Since the cloning of *Hsf* genes in tomato (*Lycopersicon esculentum* L.) [12], the *Hsf* gene family has been identified in many plant species. For example, 21 *Hsf* genes have been identified in Arabidopsis, 78 in wheat (*Triticum aestivum* L.) [13,14], 25 in maize (*Zea mays* L.) [15], 25 in rice [16,17,18], 35 in *Brassica oleracea* [19], and 29 in buckwheat (*Fagopyrum tataricum* L.) [20]. Although the number and the sequence size of Hsf proteins vary among species, their protein structures are conserved [16]. Based on their sequence structures, plant Hsfs were categorized into three classes (A, B, and C) and could be further divided into several subclasses [9,21]. Class A Hsfs contains a DNA-binding domain (DBD), oligomerization domain (OD), nuclear localization/export signals (NLS and NES), and transcriptional activation domains (AHA motifs) [6,9]. However, the Hsfs of classes B and C lack the AHA domains, and they have no evident function as transcriptional activators [6]. Moreover, the C terminus of class B Hsfs contains a highly conserved -LFGV- tetrapeptide, and previous research suggests that it functions as a repressor domain [22]. Hsfs are known to recognize and bind to the promoter sequences of many heat-induced genes that contains heat shock elements (HSE, palindromic sequence of nGAAn) to regulate their expression [6,17,23]. 

Recent studies indicate that Hsfs are involved in the response to various environmental stresses, such as heat, salt, cold, and drought challenge [24,25,26,27]. HsfA1s have been identified as master regulators of the heat stress response in Arabidopsis and tomatoes [28,29,30]. Four homologs of *HsfsA1s* genes (*AtHsfA1a*, *AtHsfA1b*, *AtHsfA1d*, and *AtHsfA1e*) have been reported in Arabidopsis, and single, double, or triple knockout of these genes have no marked effect on the HSR and the long-term thermotolerance level of Arabidopsis [31]. Over-expression of *LpHsfA1a* in tomatoes increases the acquired thermotolerance [29]. It is well known that AtHsfA2 serves as a transcriptional activators for the high-level induction of HSR, playing an important role in acquired thermotolerance in Arabidopsis [32]. *AtHsfA3* and *OsHsfA3* exhibited drought- and heat-induced expression [33,34,35]. AtHsfA5 functions as a specific repressor of *AtHsfA4* [36], and AtHsfA6a is reported as a transcriptional activator which is involved in the ABA-dependent signaling pathway in Arabidopsis [37]. *AtHsfA9* is highly expressed during seed development and contributes to embryogenesis and seed maturation in sunflowers and Arabidopsis [38,39]. Most of class B Hsfs have a repressor domain at the C-terminus and lack activator functions. AtHsfB1 and AtHsfB2b are transcriptional repressors that repress the expression of heat shock-responsive genes in Arabdopsis [9,40]. However, OsHsfB4d binds to the promoter and activates the expression of *OsHsp18.0-CI* gene expression in rice [41]. However, AtHsfB1 and AtHsfA1a could form a co-activator complex and regulate target gene expression in tomatoes [42]. Apart from participating in various stress responses, Hsfs were also found to be involved in the regulation of plant growth and development. The root hair and lateral root of *OsHsfA7* over-expression rice were shorter than those of the wild-type, indicating that *OsHsfA7* plays an important role in root growth and development [43]. In Arabidopsis, *AtHsfB4* controls the asymmetry of root stem cell divisions. Previous studies have reported that the *AtHsfB4* mutant (*scz-2*) is defective in *AtHsfB4* expression and exhibits a short root [44,45,46]. Seedlings of *hsfb1-1 hsfb2b-1* double mutant Arabidopsis possess longer hypocotyls than wild-type seedlings under normal growth conditions [22], and *AtHsfB2a* is required for plant fertility gametophyte development [47]; in tomatoes, during microsporogenesis, *LpHsfA2* plays a role in reproductive tissue development [48]. 

The *Hsf* family has been extensively studied in Arabidopsis; however, the functions of most *Hsf* family genes in crop species have not been fully elucidated. Members of the *Hsf* family have been identified in many crop species, but the lack of sufficient information has hindered in-depth studies on the stress resistance mechanisms related to *Hsfs*. Twenty-five typical *Hsfs* members have been identified in rice, and the expression patterns, genomic organization, and evolutionary processes of the *Hsf* family have been studied in rice [18,49]. The functions of most *Hsfs* in rice are still not known. 

In this study, a genome-wide analysis of the *OsHsf* gene family was conducted. The phylogenetic relationships, collinearity, functional annotation, and gene expression profiles of the *OsHsf* family were determined. The results of the phylogenetic studies provide a basis for understanding the function and evolutionary history of the *OsHsf* gene family. In addition, two drought-responsive genes, *OsHsfB4b* and *OsHsfB4d*, were identified using a public database and transcriptional expression analysis, and a subcellular localization analysis of these two proteins was performed. The function of *OsHsfB4b* was further characterized using transgenic Arabidopsis lines. Our study may facilitate subsequent studies of the evolutionary history and biological functions of the *OsHsf* gene family in rice, as well as reveal the potential value of *OsHsfB4b* in rice genetic stress adaptation improvement.

## 2. Results

### 2.1. Phylogenetic Analysis of Hsf Families in Different Species

**Figure 1:**
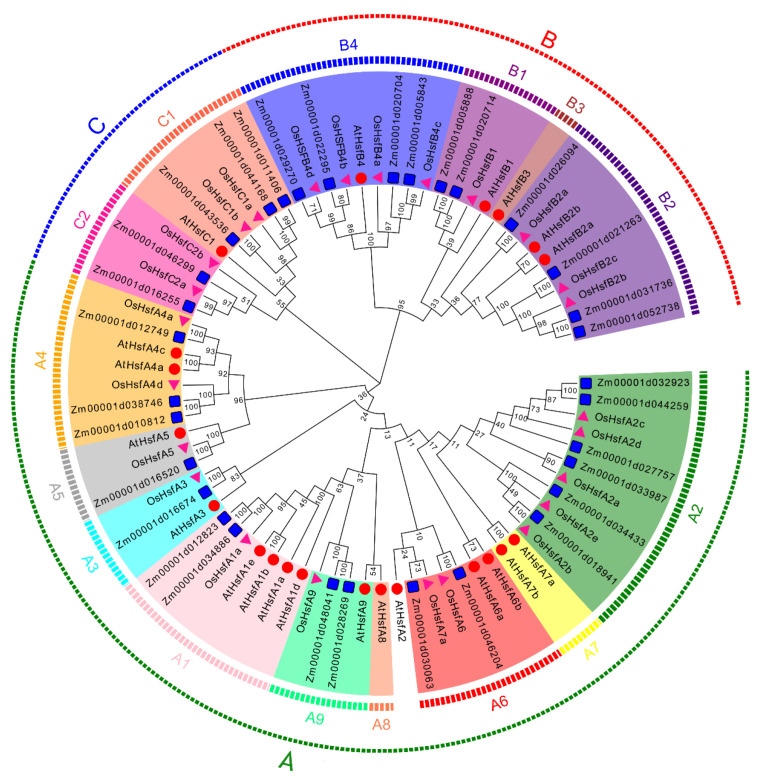
Complex phylogenetic tree of Hsf proteins in rice (*Oryza sativa* L. Os), Arabidopsis (*Arabidopsis thaliana* L. At), and maize (*Zea mays* L. Zm). The tree was generated using the protein sequences of rice (pink triangle), Arabidopsis (red circle), and maize (blue square). The tree shows three major groups (A–C) and 15 subgroups with different colored backgrounds.

The hidden Markov model of DBD domain (Pfam: PF00447) specific for the Hsf protein was utilized to screen rice Hsf proteins. Candidate proteins were further identified using the NCBI Batch CD-search and Pfam databases. Combined with previous studies [16,18], a total of 25 OsHsf proteins were obtained. Detailed information regarding the identified *OsHsf* genes is provided in Table S1. To investigate the phylogenetic relationships of the *Hsf* gene in rice (monocot model plant), maize (monocot model plant), and Arabidopsis (dicot model plant), phylogenetic analysis was performed. The phylogenetic tree showed that the Hsf proteins could be classified into three groups (A, B, and C) (Figure 1), and further divided into 15 subclasses according to bootstrap values and phylogenetic relationships. Previous studies have confirmed that because of gene duplications in the monocot lineage, the *HsfC* group in monocots is more complex than in dicots [9]. Compared with one C class *Hsf* gene in Arabidopsis, there were four *HsfC* class members in rice and five in maize. In addition, *HsfA7*, *HsfA8*, and *HsfB3* were found only in Arabidopsis. These results imply that the different evolutionary patterns in Arabidopsis, rice, and maize may occur after their divergence. 

### 2.2. Conserved Motifs of OsHsfs

To further explore the information on OsHsfs, the conserved motifs of OsHsf protein were analyzed using the NCBI conserved domain database (Figure S1). Analysis of the protein domains revealed that all OsHsf proteins contained the HSF conserved domain, which is important for OsHsfs protein functions. To further explore the potential function of OsHsf proteins, we performed conserved motif analysis using the MEME online tool (Figure S2). Motif 1 was found in all OsHsf proteins, suggesting that these regions are vital for OsHsf protein function.

### 2.3. Chromosomal Localization and Synteny Analyses of OsHsf Genes

**Figure 2:**
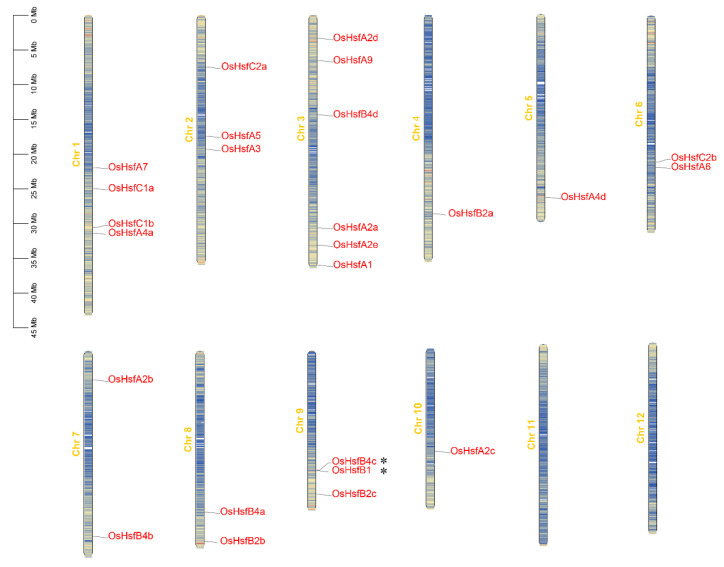
Schematic representations for chromosomal localization and gene duplication events of *OsHsf* genes. Twenty-five *OsHsf* genes were unevenly mapped on the 10 chromosomes. The lines represent the gene density in each chromosome. * indicated the tandem duplication events of *OsHsfs*.

To further understand the evolutionary history and gene expansion of *OsHsf* family genes, 25 *OsHsf* genes were mapped to rice chromosomes. The results showed that 25 *Hsf* genes were randomly located on 10 chromosomes, and the distribution positions and numbers of these genes were not uniform (Figure 2). There were no *OsHsf* genes on chromosomes 11 and 12. In terms of their overall distribution, chromosome 3 contained the most *OsHsf* genes (6), whereas the other chromosomes contained 0 to 4 genes. Tandem gene duplication events often occur during plant evolution, resulting in the expansion of gene families [50]. In this study, two *OsHsf* genes, *OsHsfB4c* and *OsHsfB1*, were clustered as tandem duplication event regions. The results indicated that gene duplication events played a driving role in the evolution of the *OsHsf* family. 

**Figure 3:**
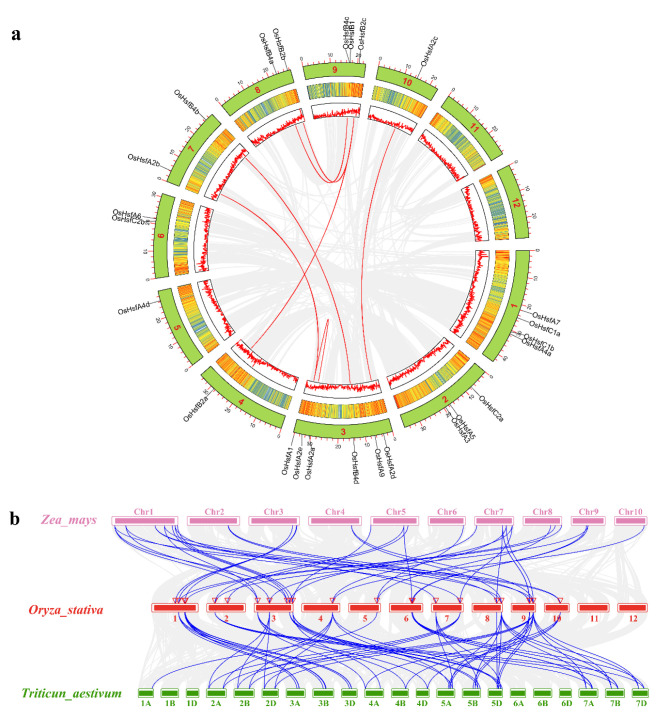
Synteny analysis of *OsHsf* genes. (**a**) Interchromosomal relationships of *OsHsf* genes. Grey lines indicate all synteny blocks of rice, and red lines indicate links beween *OsHsf* syntenic genes; (**b**) Synteny analysis of *OsHsf* genes between maize, rice, and wheat. Red triangles indicate the positions of *OsHsfs*. All collinearity blocks and collinearity blocks of *Hsf* gene pairs within rice, maize, and wheat are indicated by grey lines and blue lines, respectively.

In addition to tandem gene duplication events, segmental gene duplication is another driving force for gene family expansion [51]. BLASTP, MCScanX, and other software were used to identify seven segmental duplication *OsHsf* gene pairs (Figure 3a). These results suggest that segmental duplication and gene tandem events are two driving forces for *OsHsf* family evolution and expansion.

### 2.4. Collinearity Analysis of Hsf Family Genes in Different Species

The collinearity of gene families can provide a basis for understanding the evolutionary history of gene families in different species. A collinearity map associated with wheat, maize, and rice was constructed to further study the collinearity of the Hsf family in the representative crop species (Figure 3b). The results showed that the *OsHsf* genes had 40 homologous pairs in the wheat genome (including 20 *OsHsf* genes) and 33 homologous gene pairs in the maize genome (including 25 *OsHsf* genes). Homologous family gene members from different species were usually gathered in a group [52]. Some collinear pairs (containing 20 *OsHsf* genes) were present in both wheat and maize, suggesting that these homologous genes may have existed before the differentiation of the ancestral species. Additionally, most *OsHsf* genes had three homologous pairs in wheat and two homologous pairs in the maize genome; however, we found that *OsHsfB2c* had nine homologous gene pairs in wheat and three homologous gene pairs in maize, indicating that the *OsHsfB2c* gene was critical for the evolution of the *OsHsf* gene family. Interestingly, five *OsHsfs* genes had no homologous gene pairs in wheat. Several homologous gene pairs (containing 20 *OsHsf* genes) were found between rice and wheat, and between rice and maize, which indicated that these orthologous pairs were formed before the ancestral divergence of monocots. These results provide a basis for further understanding the developmental mechanism of the *Hsf* family in rice.

### 2.5. Cis-Elements Analysis of Hsf Gene Promoters in Rice

The promoter regions of genes usually contain many cis-acting elements that participate in various pathways [53]. Cis-element analysis can shed light on gene functions. Previous studies have confirmed that some *Hsf* genes can be induced by various stresses and phytohormones [13,49,54,55]. To further understand the cis-element information of rice *Hsf* gene promoters, the 2 kb 5′ upstream region of the 25 *OsHsf* genes was analyzed using the PlantCARE database. The results showed that, in addition to some basic core components, *OsHsf* genes contained a series of cis-elements such as ABA response element (ABRE), G-box (Sp1), MeJA response element, anoxic or anaerobic induction element, SA response element, GA response element, auxin response element, and photoperiod related response element (Figure S3). These elements are important components of abiotic stress response. In addition, the promoters of *OsHsfA1* and *OsHsfA2a* genes specifically contained a meristem expression related element, suggesting that these genes may be involved in meristem development. Furthermore, transcription factor binding sites, and MYB binding sites, were also found in some *OsHsf* promoter regions, suggesting that the *OsHsf* gene may be regulated by the MYB transcription factor. In general, the results indicate that the promoters of *OsHsf* family genes have multiple cis-elements, which may be affected by a variety of factors, and some *OsHsf* genes may be target genes for transcription factors such as MYB.

### 2.6. Functional Annotation Analysis of OsHsf Genes

To identify the potential functions of the *OsHsf* genes, the Gene ontology (GO) term enrichment analysis was performed. According to GO enrichment, *OsHsf* family genes were classified into three main categories, cellular component (CC), molecular function (MF), and biological process (BP) (Figure S4). The most significant MF GO terms for *OsHsf* family genes were DNA-binding transcription factor activity, transcription regulator activity, and identical protein binding, consistent with the fact that Hsfs are transcription factors. In addition, the main CC GO term for *OsHsf* family genes was the nucleus, and the main MP GO terms were regulation of transcription, DNA-templated, regulation of nucleic acid-templated transcription, and regulation of RNA biosynthetic processes.

### 2.7. Expression Pattern Analysis of OsHsf Genes

**Figure 4:**
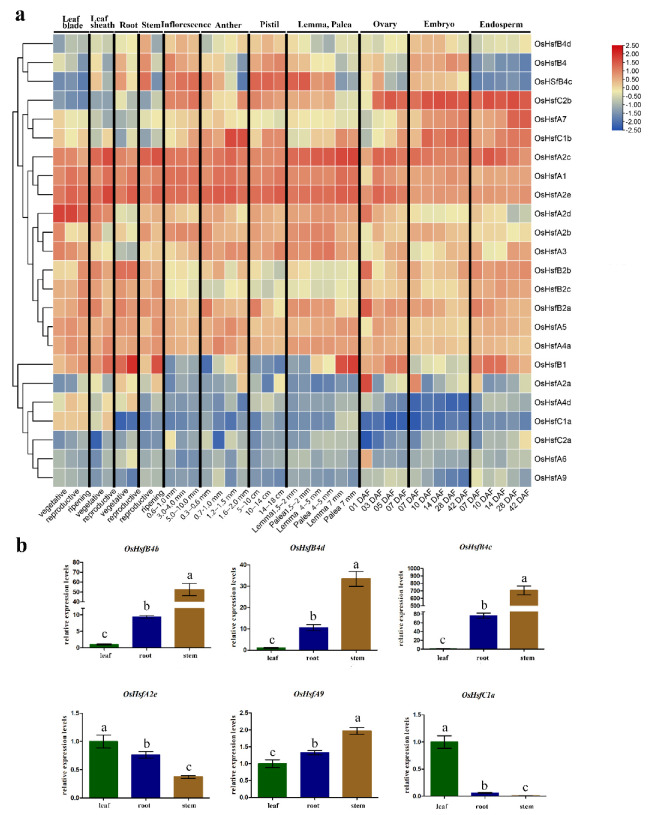
Expression profiles of the *OsHsf* genes. (**a**) Hierarchical clustering of expression profiles of *OsHsf* genes in 48 samples including developmental stages of different tissues. DAF: day after fertilization; (**b**) Expression analysis of some *OsHsf* genes in different tissues (leaf, root, and stem) by qRT-PCR. Data were normalized to *OsActin1* and *OsActin2*. Different letters indicate significant differences at *p* < 0.05 according to one-way ANOVA and post-hoc Tukey’s test.

The expression of some *Hsf* genes in Arabidopsis is tissue- or organ-specific [9]. To investigate the temporal and spatial transcription patterns of *OsHsf* genes in the rice life cycle, the expression patterns of *OsHsf* genes in 48 samples of transcriptome data from different tissues at different developmental stages of rice were selected for microarray analysis, and the expression patterns of *OsHsfs* were obtained (Figure 4). The expression pattern analysis results revealed that *OsHsfA4d*, *OsHsfC1a*, *OsHsfC2a*, *OsHsfA6*, and *OsHsfA9* genes showed low expression levels in all samples whereas *OsHsfA2c*, *OsHsfA1* and *OsHsfA2b* were highly expressed in all samples. *OsHsfA2d*, *OsHsfA2b*, *OsHsfA3*, *OsHsfB2b*, *OsHsfB2c*, *OsHsfB2a*, *OsHsfA5*, and *OsHsfA4a* were clustered into a subgroup with moderate expression levels in all tested samples. Some genes showed tissue specific expression patterns; for example, *OsHsfB4b*, *OsHsfB4d,* and *OsHsfB4c* were highly expressed in the pistil, inflorescence, and embryo. Furthermore, the expression data revealed that some duplicated *OsHsf* gene pairs (*OsHsfB4b*/*B4d*, *OsHsfB2a*/*2C*, *OsHsfB4a*/*B4c*, and *OsHsfB2b*/*B2c*) exhibited similar expression patterns between the two members, but other gene pairs and gene trios did not (*OsHsfA2c*/*A2d*, *OsHsfA2a*/*A2e*/*A2b*). This suggests that the functions of some duplicated *OsHsf* gene pairs changed during long-term evolution. 

### 2.8. Expression of OsHsfB4b and OsHsfB4d under Abiotic Stress

**Figure 5:**
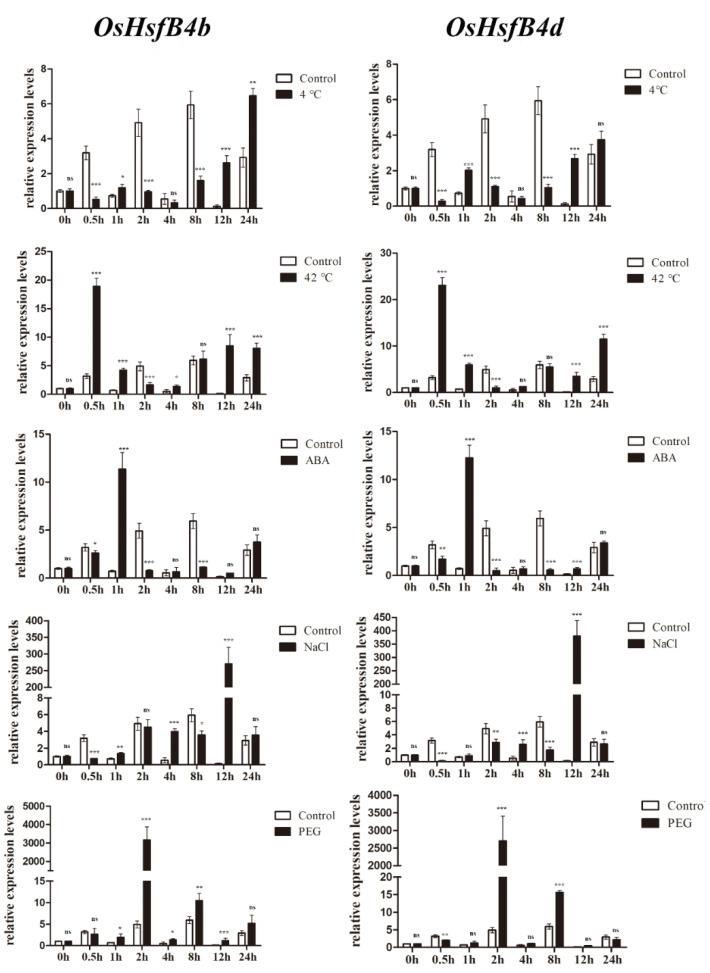
Expression levels of *OsHsfB4d* and *OsHsfB4d* in response to cold, heat, NaCl, ABA, and PEG treatments. Relative expression levels of *OsHsfB4b* and *OsHsfB4d* in response to drought for 1 h to 24 h in the leaves at the three-leaf stage of rice. Data were normalized to *OsActin1* and *OsActin2*. Values were the mean ± standard deviation of three biological replicates. ANOVA and Tukey’s test (ns inditated non-significant. * p < 0.05, ** p < 0.01, *** p < 0.001).

There are relatively few studies on the function of the class B Hsfs in rice. To investigate whether the expression of *OsHsfB4b* and *OsHsfB4d* was affected by abiotic stress and ABA treatment, 14-day-old rice seedlings were subjected to a low temperature (4 °C), high temperature (42 °C), drought (20% PEG 6000), salt (200 mM NaCl), and abscisic acid (100 μM ABA) treatments. RNA was extracted at different time points and RT-qPCR experiments were conducted to analyze the gene expression patterns under different treatments. Analysis of the expression patterns revealed that *OsHsfB4b* and *OsHsfB4d* showed similar expression patterns (Figure 5). Both *OsHsfB4b* and *OsHsfB4d* were upregulated by heat, ABA, NaCl, and PEG treatments; however, under low-temperature treatment, in comparison with the control group, *OsHsfB4b* and *OsHsfB4d* genes showed a reduction in their expression levels at 0–8 h but increased after 24 h of treatment. Under high-temperature treatment, *OsHsfB4b* and *OsHsfB4d* genes were induced quickly and peaked after 0.5 h of treatment, and then the transcript levels decreased gradually. In the presence of ABA and PEG, *OsHsfB4b* and *OsHsfB4d* were quickly induced and peaked after 1 h or 2 h of treatment, and then decreased gradually. The expression of *OsHsfB4b* and *OsHsfB4d* was strongly induced by NaCl, and peaked after 12 h of treatment. The expression patterns of *OsHsfB4b* and *OsHsfB4d* under different treatments implied that they play important roles in the response to stress conditions and signal transduction. The reliability of the transcriptome data was further validated by qRT-PCR experiments, which were carried out on three representative samples for six selected *OsHsf* genes.

### 2.9. Subcellular Localization Analysis of OsHsfB4b and OsHsfB4d 

**Figure 6:**
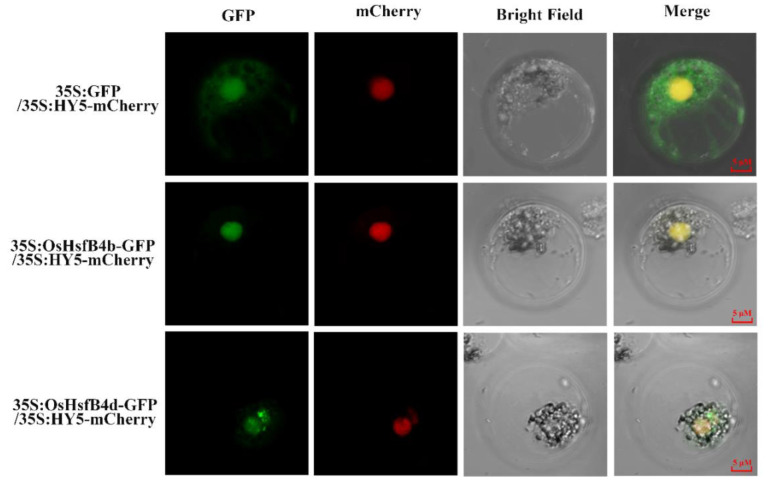
Subcellular localization of OsHsfB4b and OsHsfB4d proteins in rice protoplast. OsHsfB4b-GFP and OsHsfB4d-GFP fusion protein driven by the 35S promoter were transformed into the rice protoplast. GFP and mCherry signals are represented by a green and red color, respectively. Scale bars = 5 μM.

To further understand the subcellular localization of OsHsfB4b and OsHsfB4d, AtHY5-mCherry, OsHsfB4b-GFP, and OsHsfb4d-GFP fusion vectors driven by the 35S (CaMV 35S) promoter were transferred into rice protoplasts. The *AtHY5* gene of *Arabidopsis* was used as a nuclear marker gene, and the empty 35S: GFP vector was used as a negative control. Fluorescence signals of the transformed rice protoplasts were observed using laser scanning confocal microscopy. As shown in Figure 6, the GFP signals of 35S-GFP spread throughout the whole cell, but the fluorescence signal of OsHsfB4b-GFPcompletely overlapped with that of the nuclear marker HY5-mCherry, indicating that the OsHsfB4b protein was highly expressed in the nucleus. However, the fluorescence signal of OsHsfB4d-GFP was observed not only in the nucleus, which completely overlapped with the HY5-mCherry signal, but also in the cytoplasm near the nucleus. The similarity of OsHsfB4b and OsHsfB4d protein sequences was 61.06%, and the sequence alignment result was shown in Figure S8. However, the subcellular localization of these two proteins requires further characterization using other assays. 

### 2.10. Over-Expression of OsHsfB4b Gene in Arabidopsis and Rice Increases Drought Resistance

**Figure 7:**
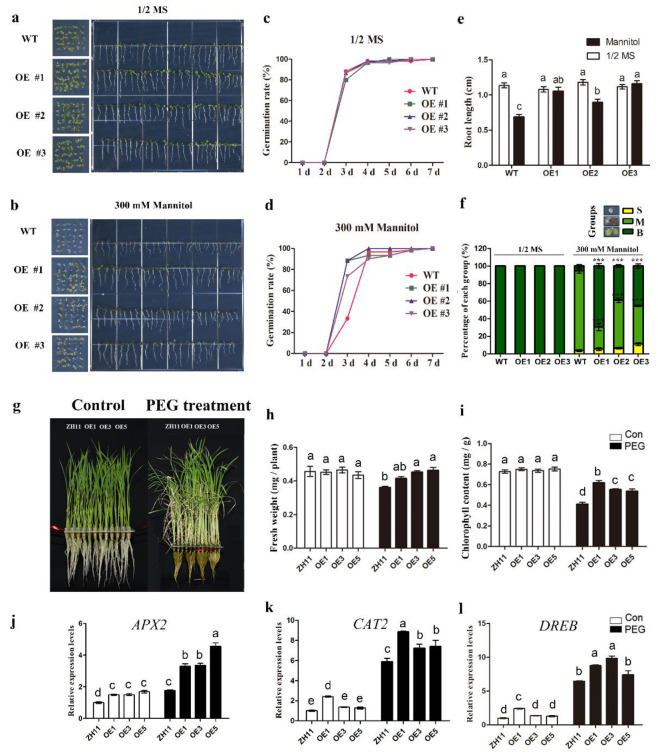
Over-expression of the *OsHsB4b* gene in Arabidopsis and rice enhances plants resistance to drought stress. Photographs of 7-day-old WT and *OsHsB4b*-over-expressed Arabidopsis seedlings grown on 1/2 MS (**a**) and 1/2 MS containing 300 mM Mannitol (**b**); The germination rates (**c**,**d**) and root lengths (**e**) of seedlings in (**a**,**b**); (**f**) Percentage of B (big), M (moderate), and S (small) seedlings in (**a**,**b**). According to the one-way ANOVA and post-hoc Tukey’s test, the significant differences were indicated by different letters in (**e**), n ≥ 40. Statistical comparisons were performed by two-tailed independent sample *t*-test (*** p < 0.001) in (**f**); (**g**) Photographs of WT and OE rice plants exposed to 20% PEG treatment. Fourteen-day-old rice seedlings were treated with 20% PEG 6000 for 8 days followed by recovering for 7 days. Thirty plants per line were used per replicate; (**h**,**i**) The fresh weight (**h**) and the chlorophyll content (**i**) of rice seedlings in (**g**); (**j**–**l**) The expression levels of *APX2*, *CAT2*, and *DREB* in leaves of WT or OE rice lines upon PEG treatment. Different letters indicate significant differences at *p* < 0.05 according to one-way ANOVA and post-hoc Tukey’s test.

The potential function of *OsHsfB4b* and full-length *OsHsfB4b* CDS driven by the CaMV 35S promoter was introduced into *Arabidopsis*, and three homozygous T3 generation lines were selected for subsequent investigation. Next, the phenotypic characteristics of these lines under different stress conditions were analyzed. Seedlings grown on ½ MS medium for 5 d showed no difference between transgenic *Arabidopsis* and wild-type (Col-0) plants (Figure 7). However, when grown on a ½ MS medium containing 300 mM mannitol, the germination rate and root length of the transgenic lines were significantly higher than those of the wild-type. The seedlings were clustered into three categories, big (B), moderate (M), and small (S). Compared with the wild-type, the percentage of B seedlings in OsHsB4b-over-expressed lines was significantly increased, and M seedlings were significantly decreased in a 1/2 MS with 300 mM mannitol (Figure 7f). There was no significant difference between transgenic lines and wild-type plants grown on a 1/2 MS medium containing different concentrations of ABA (0.5, 1, and 2 μM) and NaCl (100, 150 and, 200 mM) (Figure S6). The results showed that the *OsHsfB4b* gene was involved in the drought stress response and heterologous expression of the *OsHsfB4b* gene displayed better drought tolerance than the wild-type. 

To further investigate the role of *OsHsfB4b* in drought stress in rice, we generated 13 independent transgenic rice lines that over-expressed *OsHsfB4b* driven by the 35S promoter (Figure S7). Three over-expression (OE) lines (OE1, OE3, and OE5) were selected for further analyses research. Analysis of drought stress tolerance showed that there were no significant differences in rice seedling growth between the WT (ZH11) and the OE lines under control conditions (Figure 7g). In contrast, OE lines treated with 20% PEG performed better than the WT, with greater fresh weight, higher chlorophyll content (Figure 7h,i), and higher expression levels of the drought induced genes (*CAT2*, *APX2*, and *DREB*) (Figure 7j–l). Taken together, these results indicated that *OsHsfB4b* confers tolerance to drought stress in rice.

## 3. Discussion 

*Hsf* is a gene family of transcription factors that is widely present in all plant species. The *Hsf* family genes have been identified in the whole genome of many species [9,15,19,54]. Class B Hsfs are generally considered to be negative regulators of plant responses to abiotic stresses [22,42,56]. In Arabidopsis, AtHsfB1 and AtHsfB2b inhibit the expression of Pdf1.2a and *Pdf1.2b* genes to negatively regulate pathogen resistance [22]. In addition, under salt stress, soybean HsfB2b not only inhibits *GmNAC2* expression, but also acts as a positive regulator to activate *GmC4H* gene expression and promote flavonoid synthesis [57]. OsHsfA2d (OsHSF7) functions as a high temperature receptive and responsive factor in rice plants [58]. The *Hsf* genes has also been found to participate in plant development. Studies have shown that Arabdopsis HsfB4 controls an asymmetric cell division of root stem cells, but has almost no response to stress conditions [44,45,46,59]. Previous studies have shown that OsHsfB4d can increase rice resistance to *Xanthomonas oryzae* by inducing *OsHsf18.0-CI* expression [41]. ClpB (Hsp100) is an very important stress-responsive molecular chaperone, which facilitates proper protein folding in response to external and internal stresses [7,60]. OsHsfA2c and OsHsfB4b are in involved in transcriptional regulation of the cytoplasmic *OsClpB* gene in rice [61]. OsHsfC1b positively regulates salt and osmotic stress tolerance [62]. However, the functions of some *Hsfs* in rice are not yet known. With the development of sequencing technology, the release of whole genome sequence data of multiple species enables the systematic identification and analysis of *Hsf* family genes at the genome level.

### 3.1. Identification and Characteristic Analysis of OsHsf Genes

In this study, 25 *OsHsfs* were identified in the proteome of rice by comprehensive genome analysis. Phylogenetic analysis of rice, maize, and *Arabidopsis* was performed to classify *OsHsfs* into three major classes (A, B, and C) and 12 subclasses. Phylogenetic analysis revealed divergent expansion patterns of *Hsfs* in class C. The number of C class *Hsf* members in *Arabidopsis*, maize, and rice was one, five, and four respectively, indicating that the expansion rate of the *HsfC* subfamily differed after species differentiation. It is believed that the *HsfC* group from monocots is more complex than that from eudicots [10], which strongly supports the results of our study.

Tandem gene duplication and segmental replication events are two key mechanisms of gene family expansion [51]. In this study, two *OsHsf* genes (*OsHsfB1* and *OsHsfB4c*) were clustered into a tandem duplication event (Figure 2). Collinearity analysis showed that there were seven fragment replication events in the rice *OsHsf* family genes (Figure 3a). These results suggest that tandem duplication and segmental replication events play critical roles in the evolution and expansion of the *OsHsf* gene family. Collinearity maps of the rice and two other typical crop species, wheat, and maize were constructed and 20 *OsHsf* genes showed syntenic relationships with those in wheat, followed by 25 genes in maize (Figure 3b). Interestingly, these five *OsHsfs* had no homologous gene pairs in wheat. Several homologous gene pairs (containing 20 *OsHsf* genes) were found between rice and wheat and between rice and maize, which indicated that these orthologous pairs were formed before the ancestral divergence of monocots. These results provide a basis for further understanding the developmental mechanism of the *Hsf* family in rice. The promoter regions of genes usually contain many *cis*-acting elements that participate in various pathways [53]. Previous studies have confirmed that some *Hsf* genes can be induced by various stresses and phytohormones [13,19,54,55]. Furthermore, analysis of the promoter components of *OsHsf* genes revealed that they contained some *cis*-acting elements related to stress response and, anoxic or anaerobic induction elements. Moreover, some elements of meristem expression, such as the ABA response element (ABRE), G-box (Sp1), and MeJA response element were also found. *OsHsfB4a* and *OsHsfB4d* also contained seed-specific regulatory elements. Some *OsHsf* promoters also include MYB-binding sites (MBS), suggesting that some *OsHsfs* may be regulated by MYB transcription factors.

The expression of some *Hsf* genes expressions in Arabidopsis is tissue- or organ-specific [9]. In addition, different developmental stages of different rice tissues were selected for microarray analysis, and the expression patterns of *OsHsfs* were obtained (Figure 4). Some *OsHsf* genes are highly expressed in specific tissues and periods; for example, *OsHsfB4b*, *OsHsfB4d*, and *OsHsfB4c* are highly expressed in the pistil, inflorescence, and embryo. Furthermore, the expression data revealed that some duplicated *OsHsf* gene pairs (*OsHsfB4b*/*B4d*, *OsHsfB2a*/*2C, OsHsfB4a*/*B4c, OsHsfB2b*/*B2c*) exhibited similar expression patterns between the two members, but other gene pairs did not (*OsHsfA2c*/*A2d, OsHsfA2a*/*A2e*/*A2b*). This suggests changes in the functions of some duplicated gene pairs during long-term evolution. 

### 3.2. OsHsfB4b Enhanced Drought Tolerance in Transgenic Plants

Relatively few studies have been conducted on the function of class B Hsf in rice. In this study, we detected the expression patterns of *OsHsfB4b* and *OsHsfB4d* in response to five treatments. RT-qPCR assays of *OsHsfB4b* and *OsHsfB4d* showed similar expression patterns (Figure 5). The expression of *OsHsfB4b* and *OsHsfB4d* was increased under ABA, PEG, heat, and NaCl treatments, whereas transcript levels decreased under cold treatment. However, previous studies have shown that the *AtHsfB4* gene in *Arabidopsis* has almost no response under various stress conditions [45,63], suggesting that it may be integrated into a signaling pathway not directly related to the stress response. In this study, *OsHsfB4b* and *OsHsfB4d* were found to have significant responses to high temperature, low temperature, NaCl, and other treatments, indicating that the functions of HsfB4 in Arabidopsis and rice may be different. 

Transcriptional factors are active in the nucleus and, therefore, often contain a nuclear localization signal that enables their transport to the nucleus for transcriptional activity [37]. It has been proposed that many Hsfs can travel between the nucleus and cytoplasm to regulate their transcriptional activity [64]. AtHsfA6a proteins in *Arabidopsis* can migrate from the cytoplasm to the nucleus during salt stress [37]. The ability of AtHsfA2 to bind to HSE also requires its localization to the nucleus [65]. In this study, subcellular localization analysis of the OsHsfB4b and OsHsfB4d proteins was performed in rice protoplasts. The results indicated that OsHsfB4b was localized in the nucleus whereas OsHsfB4d was found in both the nucleus and cytoplasm (Figure 6). This suggests that OsHsfB4b and OsHsfB4d may have different activities and functions.

In this study, we analyzed the drought tolerance performance of *OsHsfB4b* gene transgenic Arabidopsis and rice lines and found that over-expression of the *OsHsfB4b* gene had opposite effects under drought stress (Figure 7). However, the mechanism by which *OsHsfB4b* enhances drought tolerance in plants needs to be further explored. 

## 4. Materials and Methods

### 4.1. Identification of Hsf GENES

The nucleotide sequences, protein sequences, and the gene annotation (GFF3) files of *Triticum aestivum* (iwgsc_refseqv1.0), rice (IRGSP-1.0), maize (Zm-B73-REFERENCE-NAM-5.0), and Arabdopsis (TAIR 10) were downloaded from the EnsemblPlants database (http://plants.ensembl.org/info/data/ftp/index.html/ accessed on 11 August 2022). The hidden Markov model of DBD domain (Pfam: PF00447) specific for the Hsf protein was downloaded from the Pfam database (http://pfam.xfam.org/ accessed on 11 August 2022) and employed to search for possible homologous genes encoded in wheat, rice, maize, and the Arabdopsis genome. Subsequently, all candidate proteins were further verified using the NCBI Conserved Domain database (https://www.ncbi.nlm.nih.gov/Structure/cdd/ accessed on 11 August 2022). After removing the redundant sequences, the remaining sequences were submitted to Pfam database (http://pfam.xfam.org/ accessed on 11 August 2022) to reconfirm the presence and integrity of the DBD domain. The incorrectly proteinsequences were then manually curated. Finally, *Hsf* gene family members were obtained. The Expasy database (https://web.expasy.org/compute_pi/ accessed on 20 March 2022) was utilized to compute the isoelectric point (pI) and molecular weight (MW). The conserved motifs of OsHsfs were analysed using the MEME online software (https://meme-suite.org/meme/tools/meme/ accessed on 20 March 2022). 

### 4.2. Phylogenetic Analysis of Hsfs

To clarify the evolutionary relationships, Hsf proteins sequences of Arabdopsis, rice, and maize were aligned using MUSCLE [66]. A phylogenetic tree was constructed using MEGA-X software with 1000 bootstrap replications using the neighbor-joining (NL) method. Evolview v2 online tool (https://evolgenius.info//evolview-v2/ accessed on 20 March 2022) [67] was used to further modify the tree.

### 4.3. Chromosomal Localization and Collinearity of OsHsf Genes

By analyzing their chromosomal positions in the rice GFF3 profile, the *OsHsf* genes were mapped onto the rice chromosomes and the gene density of chromosomes were obtained. The Multiple Collinearity Scan (MCScanX) toolkit was used to identify the gene duplication. Then, the TBtools software was used to visualize the chromosomal localizations and gene duplication events [68].

### 4.4. Cis-acting Element Analysis of OsHsfs Promoters Regions 

To predict the *cis*-acting elements of *OsHsfs* promoters, 2 kb genomic DNA sequences upstream of the start codon were obtained using the Tbtools software. Then, the obtained sequences were further submitted to the PlantCARE databases (http://bioinformatics.psb.ugent.be/webtools/plantcare/html/ accessed on 20 March 2022) to analyze the various putative cis-regulatory elements. 

### 4.5. Spatiotemporal Expression Patterns of OsHsfs in Rice from RNA-Seq Data

To explore the spatiotemporal expression patterns of *OsHsfs*, published rice transcriptomic data were downloaded from the Rice Annotation Project Database (RAP-DB) (https://rapdb.dna.affrc.go.jp/ accessed on 20 March 2022) [69]. Data from 48 different developmental stages of different tissues were collected for gene expression profiling. The normalized expression levels of *OsHsf* genes were calculated by transcripts per million (TPM). A heatmap was displayed using the log2 (TPM + 1) values.

### 4.6. Plant Material, Growth Conditions, and Treatments 

Seeds of the rice cultivar Zhonghua11 (ZH11) were sterilized and germinated in distilled water for 2 days in a chamber under a photoperiod of 16 h light/8 h dark, 30 °C, 60% humidity, and light intensity of 120 μmol/m^2^/s. The seedlings were then transferred to a modified Hoagland liquid nutrient solution. After 14 days, the seedlings were exposed to different stress treatments. For heat and cold stress, seedlings were transferred to culture chambers at 37 °C or 4 °C, and transferred to Hoagland liquid nutrient solution with 200 mM ABA, 200 mM NaCl, and 20% PEG for ABA, NaCl, and PEG treatments, respectively. Leaves were collected at 0, 0.5, 1, 2, 4, 8, 12, and 24 h and stored at −80 °C. 

The *OsHsfB4b* (gene ID: Os03g0366800) gene was cloned into pCAMBIA2300-GFP vector, and the constructs were transformed to the Arabdopsis cultivar Col-0 using the floral dip method by floral dip method. T1 plants were used to check the *OsHsfB4b* gene expression level (Figure S5). Finally, three independent T3 homozygous lines were obtained. After sterilization and vernalization, the seeds of T3 transgenic and wild-type (Col-0) *Arabidopsis* were grown on 1/2 MS medium and 1/2 MS medium containing different concentrations of NaCl, ABA, and mannitol. All seedlings were grown in a chamber under a photoperiod of 16 h light/8 h dark, 22 °C, 60% humidity, light intensity: 30 μmol/m^2^/s.

### 4.7. Determination of Subcellular Localization of OsHsfB4b and OsHsfB4d in Rice Protoplast

The open reading frame (ORF) sequences of *OsHsfB4b* and *OsHsfB4d* genes, which contained the homologous arms of pAN580-GFP, were PCR-amplified from the rice cDNA of *Nipponbare,* and cloned into the XbaI/BamHI-digested pAN580-GFP vector. The nuclear localization marker gene *AtHY5* (gene ID: AT5G11260) was PCR-amplified from the cDNA of Arabdopsis cultivar Col-0 and cloned into the XbaI/BamHI-digested 35S-mCherry vector. The extraction and transformation steps of rice protoplast were done as described previously [70]. The fluorescence of the transformed protoplast were observed under a Zeiss LSM710 (Zeiss, Jena, Germany) confocal microscope. The primer sequences were listed in Table S2. 

### 4.8. Expression Analysis Using RT-qPCR

To confirm the expression patterns of *OsHsfB4b* and *OsHsfB4d* genes, the total RNA of rice leaves was isolated using RNAiso Plus (TaKaRa, 9108). The reverse transcription was performed using the PrimeScript™ RT reagent Kit with gDNA Eraser (Perfect Real Time) (TaKaRa, RR047A). RT-qPCR for examination of the expression levels of *OsHsfB4b* and *OsHsfB4d* was performed with 2× SYBR Green qPCR MasterMix II (Universal) (Sevenbio, Beijing, China) and a Roche Lightcyler480 instrument (Roche, Basel, Switzerland). The gene expression levels were analyzed with three technical replicates. The expression levels of *OsHsfB4b* and *OsHsfB4d* genes were normalized against that of *Actin1* (Os03g0718100) and *Actin2* (Os10g0510000). The primers used in gene expression analysis were designed using the NCBI Primer-BLAST tool (https://www.ncbi.nlm.nih.gov/tools/primer-blast/ accessed on 20 March 2022) and listed in the Table S2. 

### 4.9. Go Enrichment 

The eggNOG-mapper tool (http://eggnog-mapper.embl.de/ accessed on 20 March 2022) [71] was utilized to functionally annotate the *OsHsf* family genes with Gene Ontology (GO) terms in three main categories (CC, BP, and MF). Then, the diagram of GO enrichment was visualized using TBtools software.

### 4.10. Statistical Analysis 

IBM SPSS Statistics 26 (SPSS, Chicago, IL, USA) software was used to analyzed the data. Comparisons were performed according to the one-way analysis of variance (ANOVA) and post-hoc Tukey’s test. Different lowercase letters indicated significant differences (*p* < 0.05).

## 5. Conclusions

In this present study, we performed a comprehensive analysis of the *Hsf* gene family in rice. We report, for the first time, that tandem duplication and fragment replication are two important driving forces in the evolution and expansion of *OsHsf* family genes. We verified that the OsHsfB4b protein was located in the nucleus and the OsHsfB4d protein was located in both the nucleus and cytoplasm. In addition, over-expression of *OsHsfB4b* in Arabidopsis and rice can increase tolerance to drought stress. These findings provide a better understanding of the potential functional roles of *Hsf* genes in rice. However, the potential regulatory mechanisms of *OsHsfB4b* genes in drought stress require further characterization.

## Data Availability

Not applicable.

## Supplementary Materials

The following supporting information can be downloaded at: https://www.mdpi.com/article/10.3390/ijms231810830/s1.
