# Supplementary material for: OsHsfB4b Confers Enhanced Drought Tolerance in Transgenic Arabidopsis and Rice
Source: Int J Mol Sci. 2022 Sep 16;23(18):10830. doi: 10.3390/ijms231810830 (PMC9501395; doi:10.3390/ijms231810830)
Supplement: Supplementary file 1 [file ijms-23-10830-s001.zip › Supplemental Figures.pdf]

Supplementary Figures

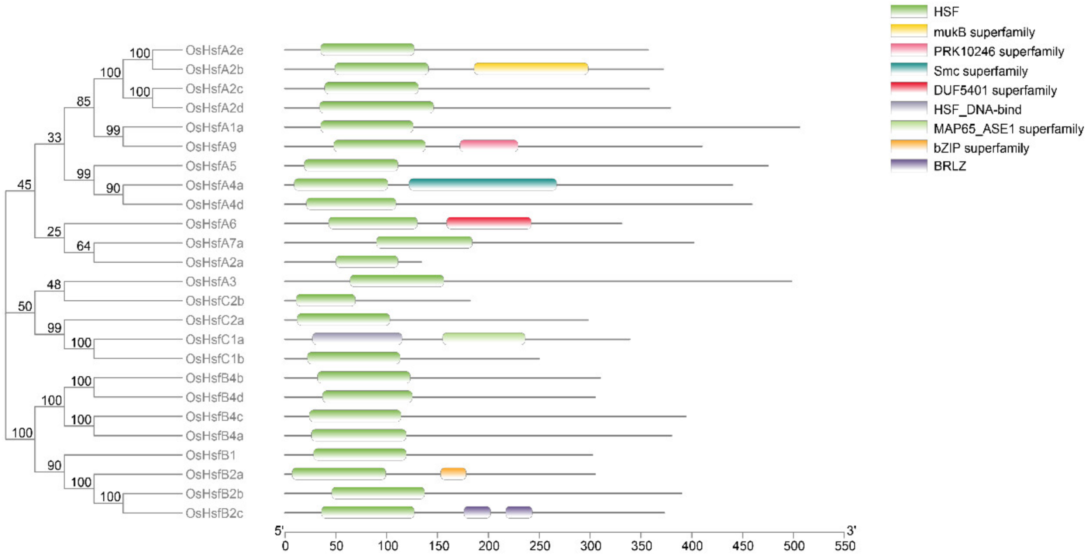

Figure S1. Schematic diagram of the conserved domain of of OsHsfs. Amino acid sequences were indicated by gray lines. Different domains were indicated by different color boxes, and each box length represents the amino acid length of the domain.

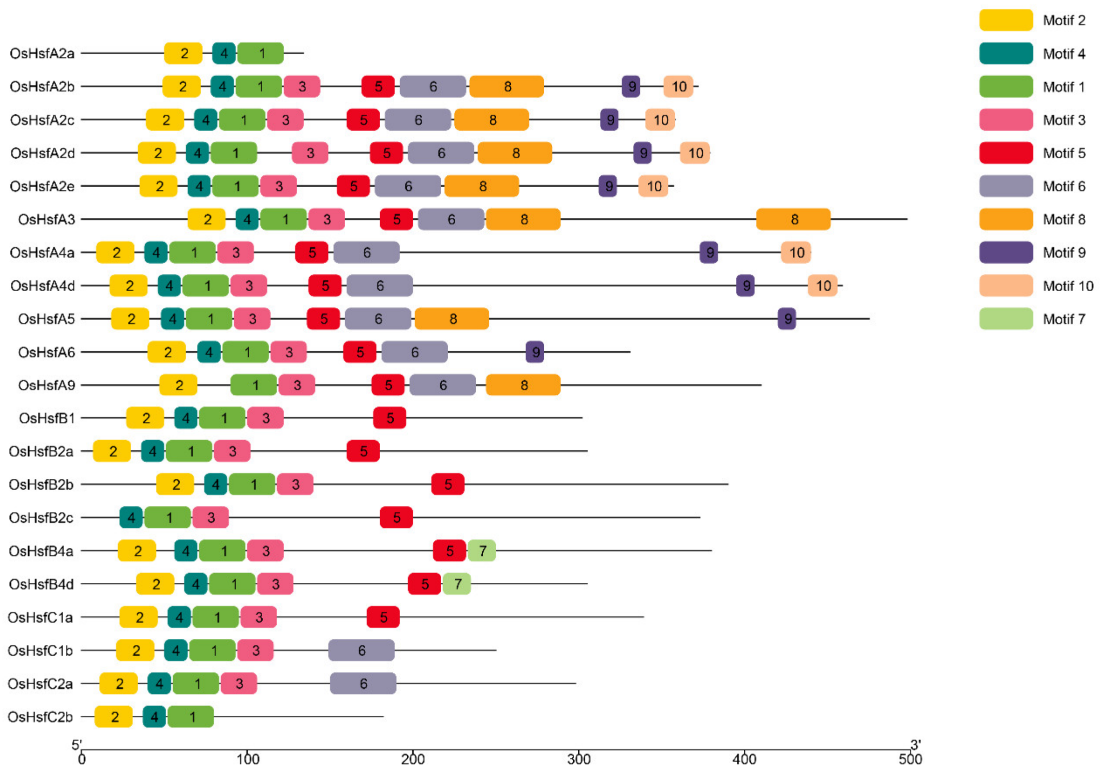

Figure S2. Distribution of conserved motifs of OsHsfs. To identify the conserved motifs, Multiple Expectation maximization for Motif Elicitation (MEME) analysis was done. Different color boxes numbered 1-10 indicated different motifs, and the annotations of motifs were listed on the right.

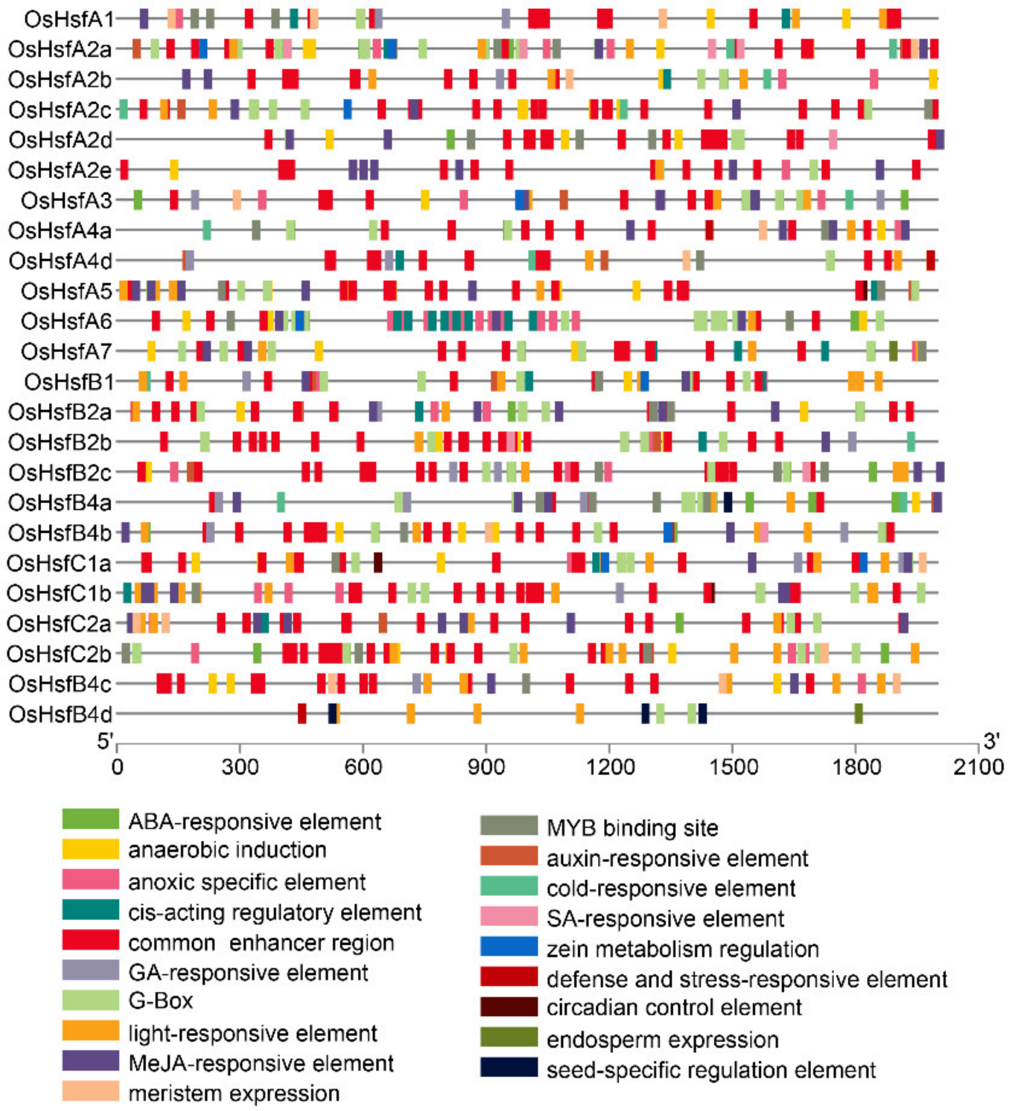

Figure S3. *Cis*-Elements in promoter regions of *OsHsf* genes. 2 kb of 5′upstream sequences of *OsHsfs* were analyzed using PlantCARE. Different elements were represented by rectangles of different colors.

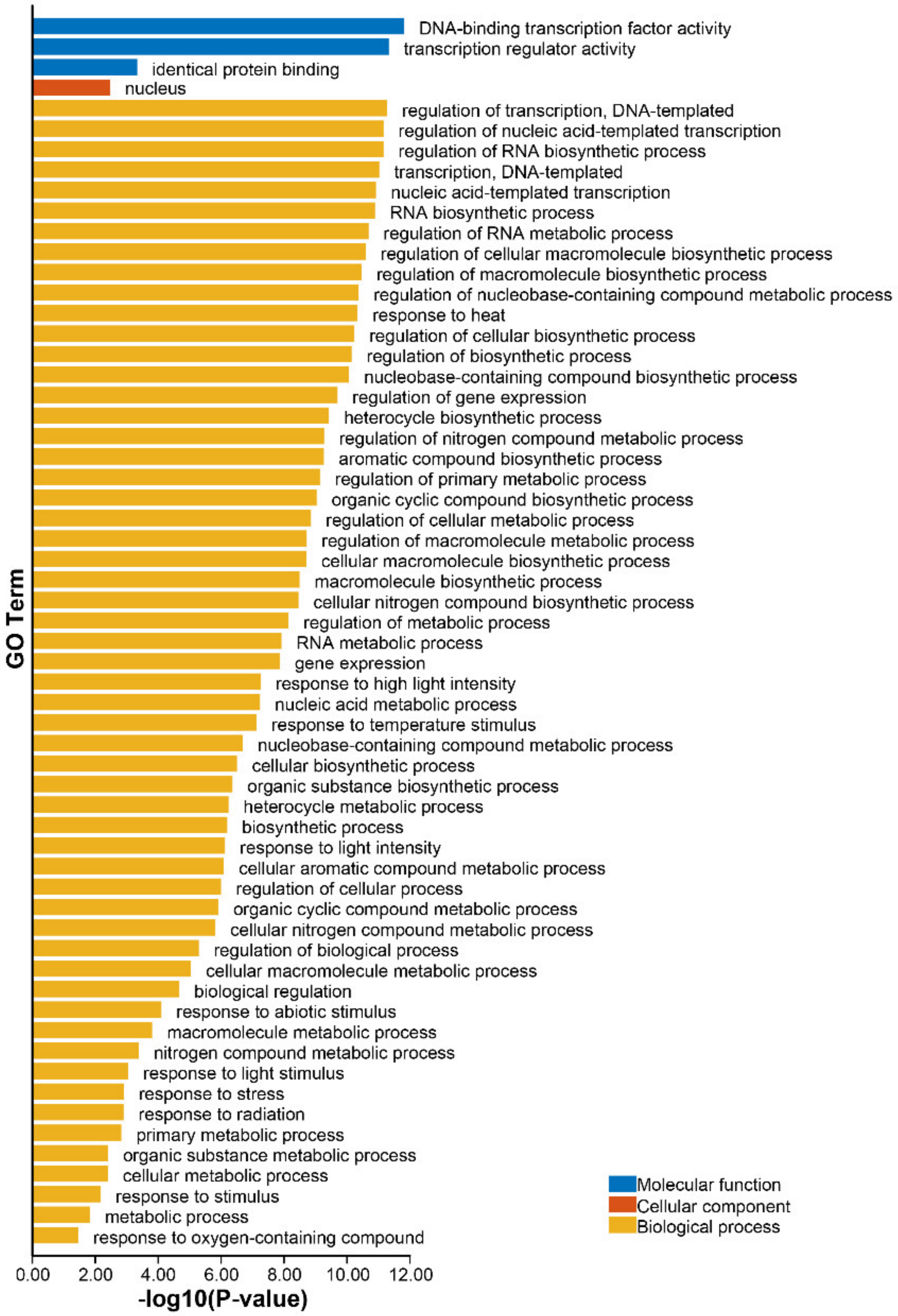

Figure S4. Gene Ontology (GO) enrichment Analysis of the *OsHsf* genes. The columns in blue, red, and orange represent the molecular function (MF), cellular component (CC), and biological process (BP) terms, respectively.

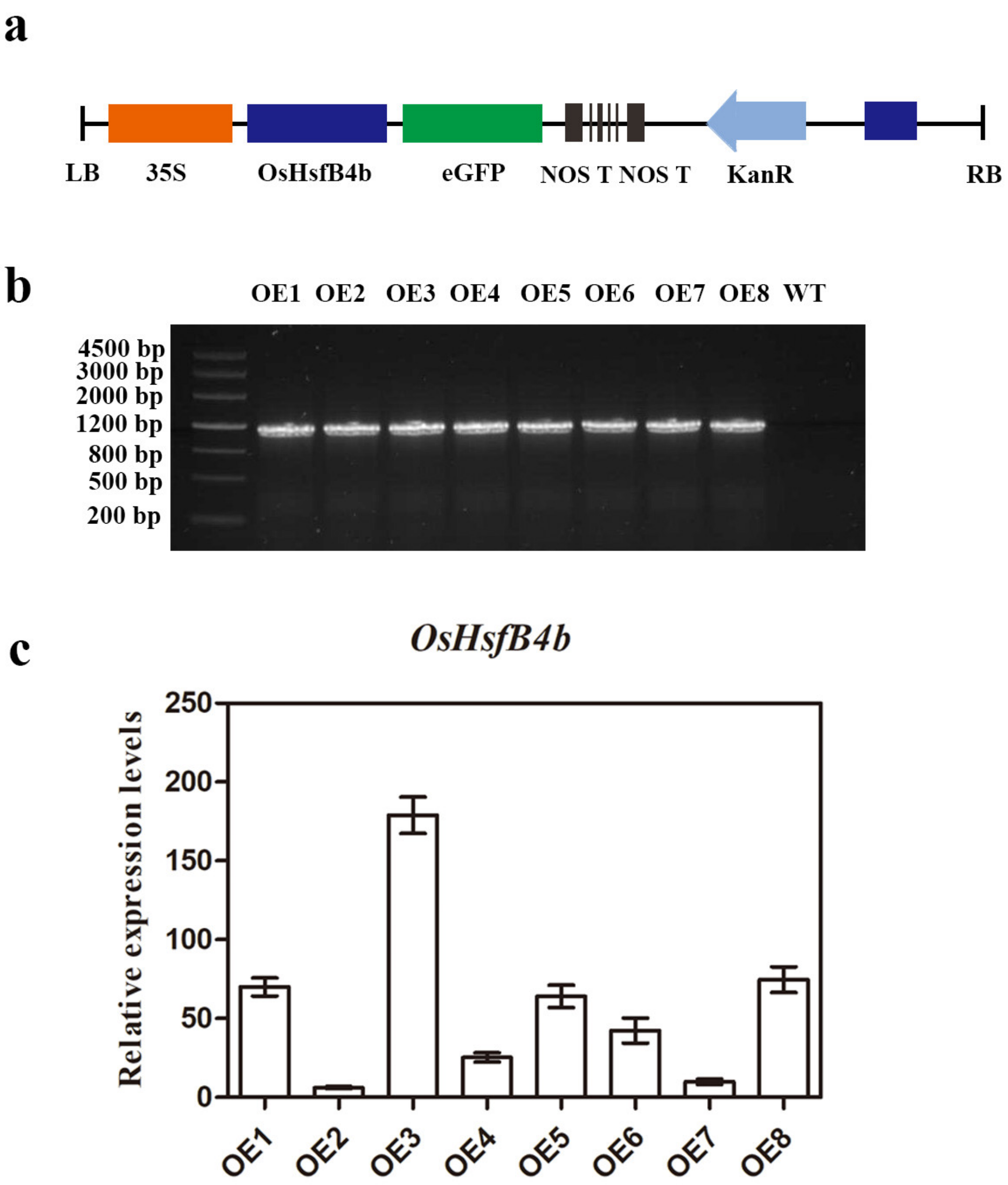

Figure S5. Identification of the *OsHsfB4b* transgenic *Arabidopsis* lines. (a) Diagram of the pCAMBIA2300-OsHsfB4b-eGFP vector. (b) PCR analysis of *OsHsfB4b* in eight *Arabidopsis* transgenic lines. (c) Relative expression levels of *OsHsfB4b* genes in eight T3 *Arabidopsis* transgenic lines, and the data were normalized with the *ACTIN 2* (AT3G18780) gene.

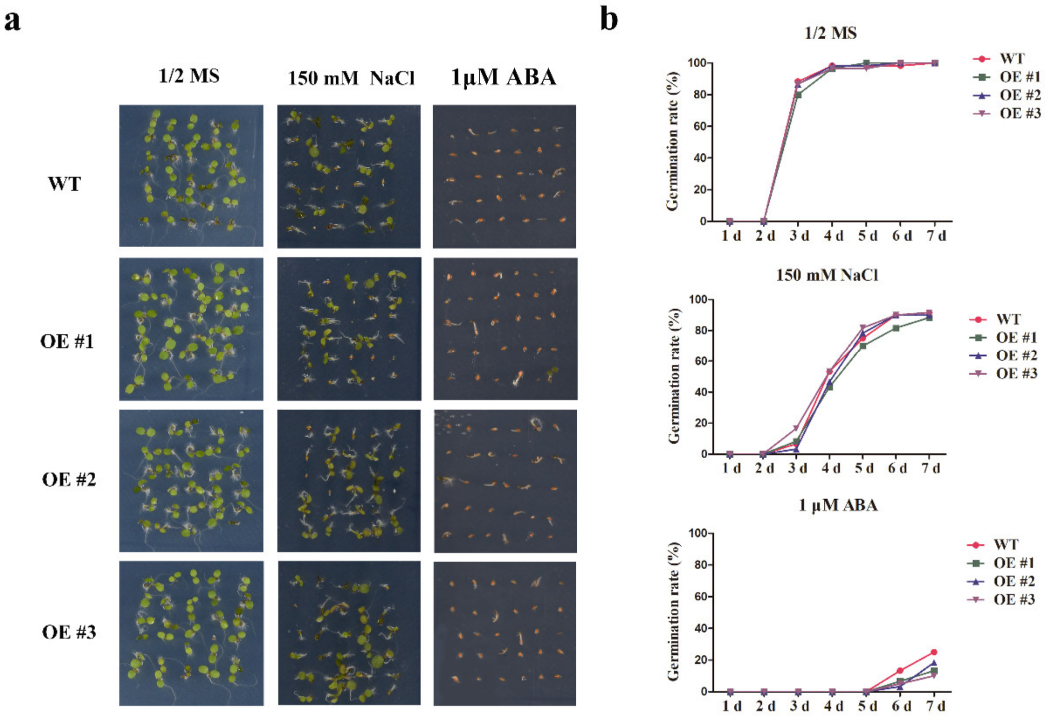

Figure S6. Phenotypes of *OsHsB4b* over-expression *Arabidopsis* under NaCl and ABA treatments. (a), Phenotypes of 7-day-old wild-type and of *OsHsB4b*-overexpressed *Arabidopsis* seedlings grown on 1/2 MS and 1/2 MS containing 150 mM NaCl and 1μM ABA. (b), The germination rates of seedlings in A, n ≥ 40.

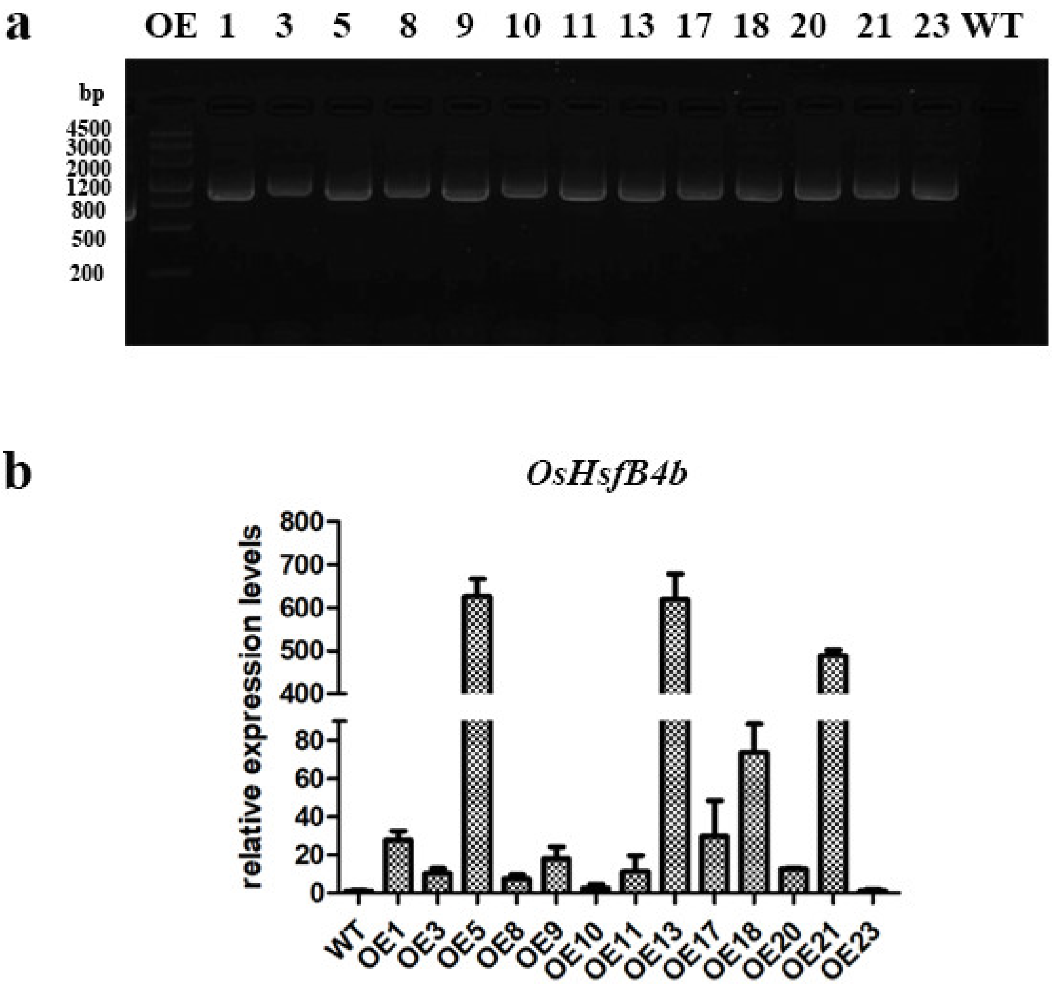

Figure S7. Identification of the *OsHsfB4b* transgenic rice lines. (a) PCR analysis of *OsHsfB4b* in 13 rice transgenic lines and WT (ZH11); (b) Relative expression levels of *OsHsfB4b* gene in 13 T3 rice transgenic lines and WT (ZH11). The data were normalized with the *OsACTIN 2* gene.

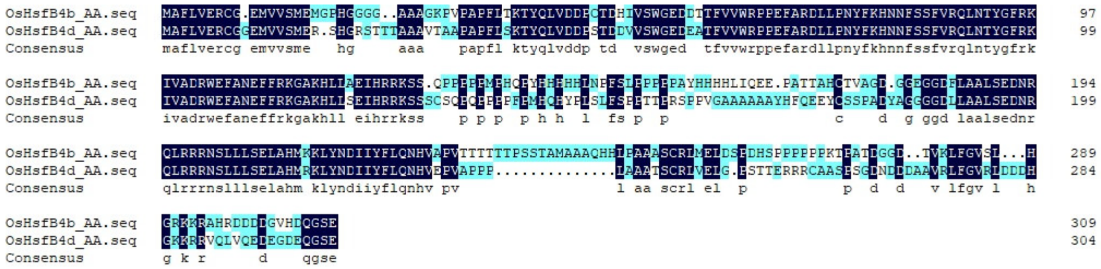

Figure S8. Amino acid sequences alignment of OsHsfB4b and OsHsfB4b.
